# The novel protein kinase C epsilon isoform modulates acetylcholine release in the rat neuromuscular junction

**DOI:** 10.1186/s13041-015-0171-5

**Published:** 2015-12-01

**Authors:** Teresa Obis, Erica Hurtado, Laura Nadal, Marta Tomàs, Mercedes Priego, Anna Simon, Neus Garcia, Manel M. Santafe, Maria A. Lanuza, Josep Tomàs

**Affiliations:** Unitat d’Histologia i Neurobiologia (UHN), Facultat de Medicina i Ciències de la Salut, Universitat Rovira i Virgili, Sant Llorenç 21, 43201 Reus, Spain

**Keywords:** PKC epsilon, Neuromuscular junction, Neurotransmission, Acetylcholine release, Electrical stimulation, Protein kinase C, Protein kinase A, Ca^2+^, Muscarinic receptors, Adenosine receptors

## Abstract

**Background:**

Various protein kinase C (PKC) isoforms contribute to the phosphorylating activity that modulates neurotransmitter release. In previous studies we showed that nPKCε is confined in the presynaptic site of the neuromuscular junction and its presynaptic function is activity-dependent. Furthermore, nPKCε regulates phorbol ester-induced acetylcholine release potentiation, which further indicates that nPKCε is involved in neurotransmission. The present study is designed to examine the nPKCε involvement in transmitter release at the neuromuscular junction.

**Results:**

We use the specific nPKCε translocation inhibitor peptide εV1-2 and electrophysiological experiments to investigate the involvement of this isoform in acetylcholine release. We observed that nPKCε membrane translocation is key to the synaptic potentiation of NMJ, being involved in several conditions that upregulate PKC isoforms coupling to acetylcholine (ACh) release (incubation with high Ca^2+^, stimulation with phorbol esters and protein kinase A, stimulation with adenosine 3′,5′-cyclic monophosphorothioate, 8-Bromo-, Rp-isomer, sodium salt -Sp-8-BrcAMP-). In all these conditions, preincubation with the nPKCε translocation inhibitor peptide (εV1-2) impairs PKC coupling to acetylcholine release potentiation. In addition, the inhibition of nPKCε translocation and therefore its activity impedes that presynaptic muscarinic autoreceptors and adenosine autoreceptors modulate transmitter secretion.

**Conclusions:**

Together, these results point to the importance of nPKCε isoform in the control of acetylcholine release in the neuromuscular junction.

## Background

Protein kinase C (PKC) regulates many neuronal functions, including ion channel activity, neurotransmitter release, membrane receptor operation and cell differentiation. The PKC family can be classified into three groups on the basis of their biochemical properties: conventional PKCs (cPKCs α, βI and βII), novel PKCs (nPKCs δ, ε, η and θ) and atypical PKCs (aPKCs ζ and ι/λ). These isoforms have distinct tissue and cell distributions [1, 2]. Intracellular PKC-binding proteins (RACKs, for receptors for activated C-kinase) achieve the specific patterns of distribution and bring activated PKC isoforms closer to their endogenous protein substrates [3, 4].

Presynaptic protein phosphorylation by the PKC family is an important mechanism that regulates transmitter release [5–9]. In the paradigmatic neuromuscular junction (NMJ), whereas protein kinase A (PKA) is tonically coupled to potentiate ACh release, PKC couples in a regulated manner when several activity demands are imposed [9–12]. The fine regulation of neurotransmission in the motor nerve terminals is modulated by presynaptic muscarinic acetylcholine autoreceptors (mAChR) [10, 13–18], adenosine receptors (AR) [19–21] and neurotrophin receptors (NR) [22–25]. Furthermore, the way that a synapse works is largely the logical outcome of the confluence of these metabotropic signaling pathways on PKC [2, 5–8]. Therefore, it is important to know which is the PKC isoform (or isoforms) that regulates acetylcholine (ACh) release in the NMJ.

Protein kinase C epsilon (nPKCε), a novel PKC isoform, is involved in regulating various cellular functions. It is highly expressed in the brain and several neural functions of nPKCε, including neurotransmitter release, have been identified [26]. nPKCε is also present in the skeletal muscle [27, 28] and it has recently been reported that nPKCε is exclusively located at the nerve terminals on the NMJ, is regulated by synaptic activity and is involved in phorbol-ester induced ACh release potentiation at the NMJ [29]. However, to date, no information is available about how the presynaptic nPKCε regulates transmitter release.

In the present study, we focused on nPKCε involvement in transmitter release. We disrupted the interaction between nPKCε and its specific RACK and therefore its activation) with an isozyme-selective translocation peptide inhibitor (εV1-2) in acute electrophysiological experiments in the adult NMJ. We observed that the nPKCε played a key role in several conditions involving PKC isoforms coupling to ACh release potentiation (for instance, incubation with phorbol 12-myristate 13-acetate –PMA-, increased Ca^2+^ inflow and PKA stimulation with Sp-8-BrcAMP -Adenosine 3′,5′-cyclic Monophosphorothioate, 8-Bromo-, Rp-Isomer, Sodium Salt-). In all these conditions, preincubation with the translocation inhibitor εV1-2 impairs PKC coupling to release potentiation. We also found that interference with nPKCε translocation and activity impedes the well known functional operation of the mAChR and AR in the control of transmitter secretion. We conclude that nPKCε is an essential element that modulates ACh release in the NMJ.

## Results

### Inhibition of nPKCε by the peptide εV1-2 in basal conditions

**Fig. 1:**
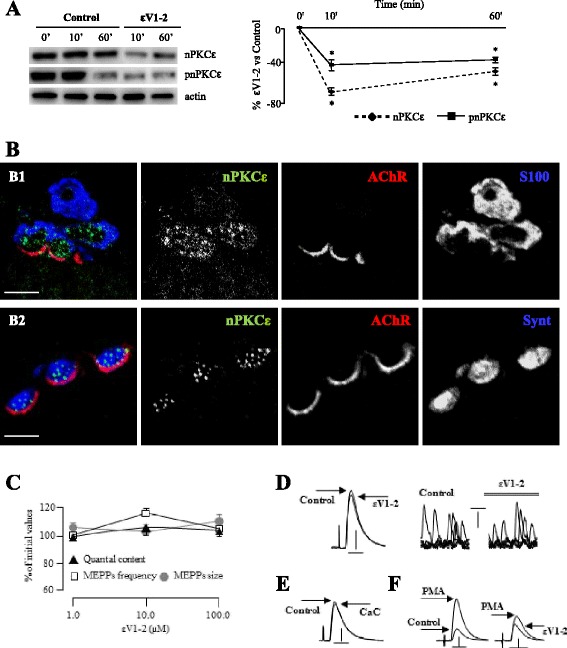
nPKCε in NMJ. **a** Western blot analysis of nPKCε and pnPKCε in the synaptic membrane of diaphragm muscles in the control and after incubation with the inhibitor peptide εV1-2 (100 μM). Western blot is shown at the left and quantitation at the right. **b** Immunohistochemistry in semithin sections from a whole-mount multiple-immunofluorescent stained LAL muscle. nPKCε isoform immunolabaled in green, AChRs in red, and the Schwann cell (S-100, in **B1**) or syntaxin (Synt, in **B2**) in blue. **c** Quantal content, MEPP frequency and MEPP amplitude in basal conditions and after 1 h of incubation with εV1-2 at concentrations of 1–100 μM. **d** Intracellular recordings show evoked EPPs and spontaneous MEPPs in basal conditions and after incubation with εV1-2. Examples of EPPs (left, horizontal bar 4 ms, vertical bar 5mv) and MEPPs (right, horizontal bar 20 ms, vertical bar 0.5mv) superimposed raw data. **e** Intracellular recordings show evoked EPPs in basal conditions and after incubation with CaC (horizontal bar 4 ms, vertical bar 5mv). **f** Raw data shows the effects of PMA in basal conditions and after incubation with εV1-2 on ACh release (horizontal bars 4 ms; vertical bars, left 10 mV, right 5 mV)

To inhibit the nPKCε activity we used an isozyme-selective translocation peptide inhibitor (εV1-2; [30, 31]) derived from the C2 domain of the nPKCε. It binds to the anchoring protein εRACK (β’COP) and disrupts the interaction between nPKCε and its specific εRACK inhibiting thus, its translocation to the membrane and so its activation. Western blot analysis was carried out to determine the presence of the nPKCε isoform in rat diaphragm skeletal muscle. Synaptic membranes were obtained as previously described [12, 27]. Fig. 1a (left and right) shows that incubation with the εV1-2 peptide (100 μM) results in a rapid (10 min) and considerable decrease in nPKCε (70 %) and phosphorilated protein kinase C epsilon (pnPKCε) (40 %) in the synaptic membrane. This initial reduction is maintained after at least 60 min of incubation with the inhibitor peptide. These changes in the level of nPKCε and pnPKCε induced by incubation with εV1-2 confirm that the peptide affects nPKCε levels. Furthermore, both, the nPKCε phosphorylation and its translocation to the membrane are indicative of nPKCε activation. Therefore, the decrease in pnPKCε in the synaptic membrane fraction indicates a less amount of active nPKCε and also indicates that the peptide is correctly acting to inhibit the action of this isoform. No change was observed in the expression of the nPKCε and pnPKCε in the presence of 100 μM of the scrambled peptide (not shown). Fig. 1b1 shows semithin cross-sections from whole-mount multiple-immunofluorescent stained levator auris longus muscles (LAL) [32] that demonstrate that nPKCε is exclusively located at the nerve terminal of the NMJ. The image shows a nPKCε fine granular green immunofluorescence located over the postsynaptic line of the nicotinic acetylcholine receptor (nAChR) site (in red) and externally surrounded by the Schwann cell (S-100, in blue). This green zone corresponds to the syntaxin (Synt) labeled axonal buttons of the nerve terminal (B2). These results all suggest that the nPKCε isoform is tonically involved in some nerve terminal mechanism because nPKCε is exclusively localized in the presynaptic component at the NMJ.

To determine whether nPKCε is constitutively involved in the mechanism of ACh release in resting NMJs, we performed electrophysiological experiments in muscles incubated with εV1-2 and carried out a concentration-dependence analysis in the range of doses commonly used in a variety of cells and models (1–100 μM, 1 h incubation). Fig. 1c shows that the different concentrations used changed neither the quantal content of the evoked endplate potentials (EPP) nor the size or frequency of the miniature endplate potentials (MEPPs) (in all cases p > 0.05). Raw data of the MEPPs (right) and EPPs (left) in the presence and absence of εV1-2 (100 μM) are shown in Fig. 1d. We also performed some experiments with εV1-2 (10–100 μM) for 3 h which had no effect on ACh release (percentage of change in the quantal content: 4.14 % ± 1.56; p > 0.05). Moreover, preincubation with εV1-2 does not change the normal depression of the EPPs (about a 50 % reduction in size) observed at 40Hz after two minutes of continuous stimulation (data not shown). Thus, the results show that there is a lack of tonic coupling to transmitter release of nPKCε in basal conditions. This result is in accordance with the well established lack of effect in basal conditions of the PKC paninhibitor Calphostin C (CaC) which acts on the regulatory domain (C1) of all PKC isoforms (Fig. 1e and also [9]). These results demonstrate that in resting muscles which do not receive action potentials from the motor neuron soma, neither PKC nor nPKCε are coupled to regulate ACh release.

However, we observed that in basal conditions, nPKCε inhibition with εV1-2 fully inhibited the well established PMA-induced pharmacologic potentiation of ACh release (Fig. 1f and also [29] indicating that nPKCε plays a role in neurotransmission at the NMJ. Therefore, it seems that although nPKCε is not involved in neurotransmission in basal conditions, this isoform plays a key role in regulating ACh release when PKC family is stimulated by PMA and coupled to the neurotransmission mechanism. In previous studies, we found several other conditions in which PKC is coupled to enhance evoked neurotransmitter release. In particular, quantal content was effectively reduced by CaC incubation (indicating the regulated coupling of PKC isoforms to ACh release) in several conditions with enhanced neurotransmission such as high Ca^2+^ media, electrical stimulation (continuous at 1Hz), PKA stimulation (with Sp-8-BrcAMP) or mAChR block or imbalance (for instance with atropine –AT–) [9–12]. Therefore, we decided to investigate the possible involvement of nPKCε in the PKC isoforms coupling to the release in these conditions.

### nPKCε in high Ca^2+^ and during continuous electrical stimulation

**Fig. 2:**
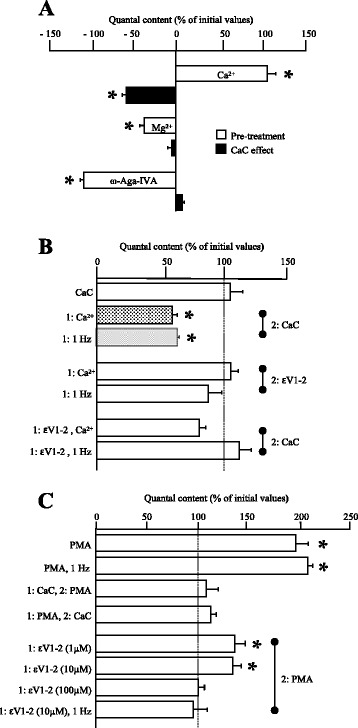
Effect of εV1-2 in high Ca^2+^ or PMA medium and during electrical stimulation on transmitter release in diaphragm muscle. **a** Histogram shows the effect of high Ca^2+^ (5 mM), high magnesium (5 mM) and the P/Q-type channel blocker ω-Agatoxin-IVA (100 nM) on the evoked transmitter release in basal conditions and after CaC incubation (2: CaC; 10 μM). The histogram in (**b**) compares the effect of CaC or εV1-2 in high Ca^2+^ medium and during continuous electrical stimulation (1Hz, 1 h) on the transmitter release. Diaphragm muscles were preincubated (1 h) with high Ca^2+^ (5 mM; 1: Ca^2+^) and then evaluated the effect of εV1-2 (10 μM, 1 h of incubation; 2: εV1-2). We also evaluated the εV1-2 effect during electrical stimulation at 1 Hz (1: 1 Hz, 2: εV1-2). To evaluate the effect of the unspecific blocker CaC when the peptide εV1-2 is present, we performed a pretreatment with εV1-2 and a second incubation with CaC (10 μM, an additional hour; 2: CaC). This was done in conditions of both high Ca^2+^ (10 μM, 1 h of incubation, 1: εV1-2, Ca^2+^) and continuous electrical stimulation (1: εV1-2, 1Hz). **c** Changes in ACh release after PMA (10 nM) and PMA in presence of continuous electrical stimulation (PMA, 1 Hz, 1 h). We also evaluated the changes in ACh release when a PMA or a CaC (10 μM) preincubated muscle was then incubated with the other drug (1: CaC, 2: PMA; 1: PMA, 2: CaC). To determine whether nPKCε affects the PMA-induced enhancing of neurotransmission, we preincubated the neuromuscular preparation (1 h) with the εV1-2 peptide (1: εV1-2, 1 μM, 10 μM, 100 μM) and then evaluated the effect of PMA (2: PMA). We also studied the link between electrical stimulation and PMA effects in presence of electrical stimulation at 1Hz (1: εV1-2, 10 μM, 1 Hz; 2: PMA). * *p* < 0.05 vs. the corresponding control

The isoform nPKCε is a novel PKC activated by diacylglycerol but not by Ca^2+^. Only classical PKC isoforms are activated by Ca^2+^. However, changes in external Ca^2+^ concentration and Ca^2+^ inflow at nerve terminals through voltage-dependent calcium channels (VDCC) lead to changes in ACh release. Fig. 2a shows increased quantal content in high Ca^2+^ (5 mM) and decreased quantal content in high Mg^2+^ (5 mM) or after the P/Q-type VDCC block with ω-Agatoxin-IVA (ω-Aga-IVA; 100 nM). The figure also shows that the increased release in high Ca^2+^ can be partly mediated by PKC activation because it is reduced by CaC whereas CaC has no effect on the low Ca^2+^ entry and low release conditions produced in high Mg^2+^ or in the presence of the P/Q-type VDCC blocker ω-Aga-IVA.

Quantal content was also reduced by CaC incubation when neurotransmission was maintained in the neuromuscular preparation by constant electrical stimulation at 1 Hz (Fig. 2b). Although there is no change in quantal content at this stimulation rate (no facilitation or depression), the effect of CaC indicates the coupling of some PKC isoforms to maintain ACh release during activity. The shadowed columns in Fig. 2b compare the similar effects of CaC at high Ca^2+^ concentrations and during continuous activity imposed at 1 Hz, two of the conditions in which PKC family is coupled to ACh release. Here we tried to know whether nPKCε plays a role controlling the involvement of other PKC isoforms in ACh release in these conditions.

Although nPKCε is Ca^2+^ independent, we performed experiments in high Ca^2+^ and during stimulation at 1 Hz to evaluate a possible involvement of nPKCε in the coupling of PKCs to transmitter release in these conditions. After a preincubation (1 h) with high Ca^2+^ (5 mM), or after a similar period of continuous stimulation, we evaluated the effect of εV1-2 (10 μM, 1 additional hour of incubation). We found no change in quantal content (Fig. 2b). These results suggest that nPKCε may not be directly related to ACh release and that CaC in high Ca^2+^ or during activity may inhibit another PKC isoform coupled to release. However, we also performed a pretreatment with εV1-2 (10 μM, 1 h of incubation) and a second incubation with CaC (10 μM, an additional hour). We worked in high Ca^2+^ media and with continuous electrical stimulation at 1 Hz. The last two columns in Fig. 2b show that, in these two conditions, CaC cannot reduce ACh release, as expected. Therefore, it seems that the effect of high Ca^2+^ and electrical activity on PKC isoforms coupling to release cannot be reversed by blocking nPKCε but can be prevented by previous nPKCε block. These results indicate the involvement of nPKCε in transmitter release by regulating the coupling of other/s PKC isoform/s, and suggest that once nPKCε has been recruited by the ACh release mechanism (in the presence of high Ca^2+^ or during the continuous electrical stimulation processes), a long-lasting function in the membrane may make the kinase competent for some time. Thus, new translocation and activation may be unnecessary.

### nPKCε in phorbol ester-induced ACh release

As showed above in Fig. 1f, evoked ACh release was strongly stimulated by PMA and this effect of PMA can be prevented by preincubation with CaC, which acts on the same domain of PKCs (C1) as PMA (Fig. 2c). However, CaC cannot reverse the effect of PMA, which indicates the potency of PMA as a positive pharmacological regulator of PKC activity. Figure 2c also shows that PMA does not need to coincide with electrical stimulation to potentiate release by about 100 % by coupling PKC isoforms. Moreover, PMA-induced potentiation was fully inhibited when εV1-2 (100 μM) was present in the media indicating that nPKCε seems to play an important role in the PKC coupling to ACh release enhancement induced by PMA. A similar role of nPKCε has been described above in high Ca^2+^ and in stimulation-induced synaptic activity conditions. Therefore, next, we investigate whether the effects of blocking nPKCε translocation in both PMA-induced and electrical stimulation-induced PKC coupling to ACh release are in any way similar or related. We performed a pretreatment with εV1-2 (1 μM, 10 μM and 100 μM, 1 h of incubation) before a second incubation with PMA (10 nM, an additional hour) under basal conditions. Then, we repeated these experiments with coincident electrical stimulation at 1 Hz. In basal conditions, PMA did not exert its full effect when εV1-2 was present at 10 μM, but the phorbol ester-induced increase in transmitter release is completely occluded after pretreatment with 100 μM εV1-2 (1 h of incubation) (Fig. 2c). Interestingly, however, the result (a full abolition of PMA potentiation) was the same when εV1-2 was present only at 10 μM but coincides with electrical stimulation at 1Hz. Thus, the tonic coupling of PKC to maintain ACh release during activity (maintenance effect) and the PMA-induced coupling that results in ACh release potentiation (potentiation effect) share a common nPKCε-based link.

### nPKCε and PKA activity

**Fig. 3:**
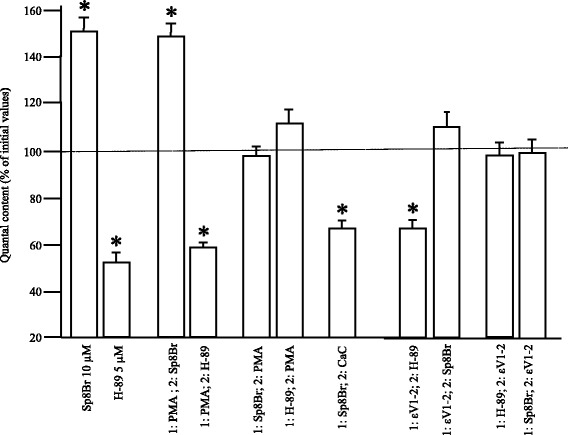
nPKCε and PKA activity in diaphragm muscles*.* Changes in ACh release after PKA stimulation (Sp-8-BrcAMPs, 10 μM) and PKA inhibition (H-89, 5 μM). Moreover, Sp-8-BrcAMPs and H-89 were incubated before and after PMA (10 nM) and εV1-2 incubation (10 μM). Seventh column shows the effect of CaC (10 μM) after a preincubation with Sp-8-BrcAMPs * *p* < 0.05 vs. the corresponding control

Some dependence of PKC on PKA activity in the fine control of neuromuscular synaptic functionalism and ACh release has also been shown [11]. Thus, the quantal content was reduced after CaC incubation (indicating the coupling of PKC to the maintenance or potentiation of ACh release) when neurotransmission was previously enhanced by PKA stimulation with Sp-8-BrcAMP [10]; see also Fig. 3 seventh column). Figure 3 gathers together some newly reproduced data of previously published results [10, 11] to facilitate comparisons with and the interpretation of the results shown in the last part of the figure. PKA inhibition in basal conditions (N-[2-((p-Bromocinnamyl)amino)ethyl]-5-isoquinolinesulfonamide, 2HCl -H-89-, 5 μM) reduces ACh release whereas stimulation (Sp-8-BrcAMPs, 10 μM) increases it. Thus, unlike PKC, PKA can be tonically active in release maintenance in basal conditions. Figure 3 also shows that PKA was able to modulate ACh release independently of PKC activity because once PKC activity had been enhanced by PMA, further stimulation or inhibition of PKA increased or decreased ACh release normally. However, once PKA had been inhibited or stimulated, PMA did not increase ACh release. It seems, then, that PKA stimulation caused PKC coupling to release, so PKC could not be further pharmacologically stimulated with PMA. This means that PKC may be reaching a maximum level of activity depending of PKA. On the other hand, PKA inhibition prevented PKC from being stimulated and coupled to ACh output.

Next, we analyzed the involvement of the nPKCε isoform in this PKA-mediated PKC activity. The inhibitor εV1-2 was added in order to block nPKCε translocation before PKA stimulation with Sp-8-BrcAMPs or inhibition with H-89 (Fig. 3). Interestingly, PKA inhibition with H-89 can reduce normal release. So PKA seems active and its coupling to ACh release can be inhibited normally by H-89 independently of nPKCε status. However, PKA stimulation with Sp-8-BrcAMPs cannot increase ACh release after preincubation with εV1-2. It seems that nPKCε translocation enables PKA to increase its coupling to ACh release and potentiate it above a basal constitutive coupling.

Activation and inhibition of PKA was also evaluated before εV1-2 pre-incubation. When nPKCε translocation is blocked after PKA stimulation with Sp-8-BrcAMPs or after inhibition with H-89, there is no change in ACh release, which suggests that nPKCε is not able to modulate neurotransmission once the mechanism of release has been activated or inhibited by the action of PKA. However, quantal content is reduced by CaC when neurotransmission is previously enhanced by PKA stimulation with Sp-8-BrcAMP, which indicates the coupling of PKC (isoforms other than nPKCε) to ACh release maintenance or potentiation (seventh column in the Fig. 3).

In summary, at this point we define four conditions that result in PKCs coupling to maintain or potentiate ACh release and in which nPKCε plays a meaningful role: i) continuous synaptic activity by electrical stimulation, ii) high external Ca^2+^, iii) direct PKC stimulation with PMA and iv) PKA stimulation with Sp-8-BrcAMPs. These four conditions involve high ACh release by increased quantal content (in the cases of high Ca^2+^, PMA and Sp-8-BrcAMPs) or merely by repeated ACh secretion events in electrical stimulation. When nPKCε is blocked with the peptide εV1-2, the PKC coupling to ACh release (which can be seen by using CaC) cannot be demonstrated in electrical stimulation and high Ca^2+^ conditions and the ACh release potentiation does not occur in PKC stimulation with PMA and PKA stimulation with Sp-8-BrcAMPs.

### nPKCε and the mAChR signaling pathway

**Fig. 4:**
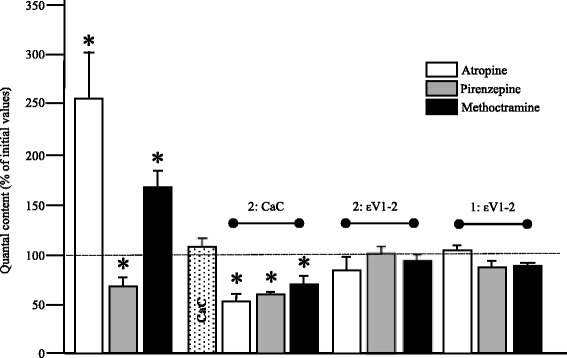
nPKCε and the mAChR signaling pathway in diaphragm muscles. Histogram shows changes in ACh release in the presence of mAChR blockers (PIR, 10 μM; MET, 1 μM; AT, 2 μM). We evaluated its effects in basal conditions and after a second incubation with CaC (2: CaC) or εV1-2 (2: εV1-2). We also preincubated with εV1-2 (1: εV1-2) and then blocked mAChR. * *p* < 0.05 vs. the corresponding control

It is known that presynaptic mAChRs can control PKC activity. Figure 4 includes some newly reproduced data of previously published results [10, 16] so that comparisons with the results involving nPKCε can be made. The figure shows the effect on ACh release of the M1 mAChR-subtype blocker pirenzepine (PIR), the M2 blocker methoctramine (MET) and the panmuscarinic blocker atropine (AT). The use of these inhibitors shows that M1-type potentiates release whereas M2-type reduces release in the adult NMJ. Moreover, when the M1/M2 mechanism is fully blocked with the unselective inhibitor AT, ACh release is potentiated which indicates a predominance of the M2 mechanism in resting conditions. The figure also shows that after blocking the muscarinic mechanism with AT – but also the M1 mechanism with PIR or the M2 mechanism with MET, thus producing an M1/M2 imbalance – PKCs become coupled to potentiate ACh release. This coupling was assessed by the reduction of release produced by CaC in these circumstances. Therefore, we studied the possible involvement of nPKCε in this mAChR-linked PKC coupling to ACh release. The inhibitor εV1-2 was added to block nPKCε translocation after or before a preincubation (1 h) with the M1 blocker PIR, the M2 blocker MET or the panmuscarinic blocker atropine AT. Figure 4 shows that the peptide makes no change when it is incubated after the blockers of mAChRs, which suggests that nPKCε translocation is not an important step in controlling release after mAChR modulation has been established. In reciprocal experiments, we evaluated the well known effect of muscarinic drugs after a εV1-2 pre-incubation. The figure shows that after an initial incubation with εV1-2 (1 h), a second incubation with AT, PIR or MET produces no change in EPP size. This indicates that (as what happens in high Ca^2+^ media, continuous electrical activity, PMA incubation and Sp-8-BrcAMPs incubation) once drug-induced muscarinic modulation has been produced on release, it is not affected if nPKCε is blocked but can be prevented by previous nPKCε translocation block. This emphasizes the importance of nPKCε allowing transmitter release control.

### nPKCε and the purinergic signaling pathway

**Fig. 5:**
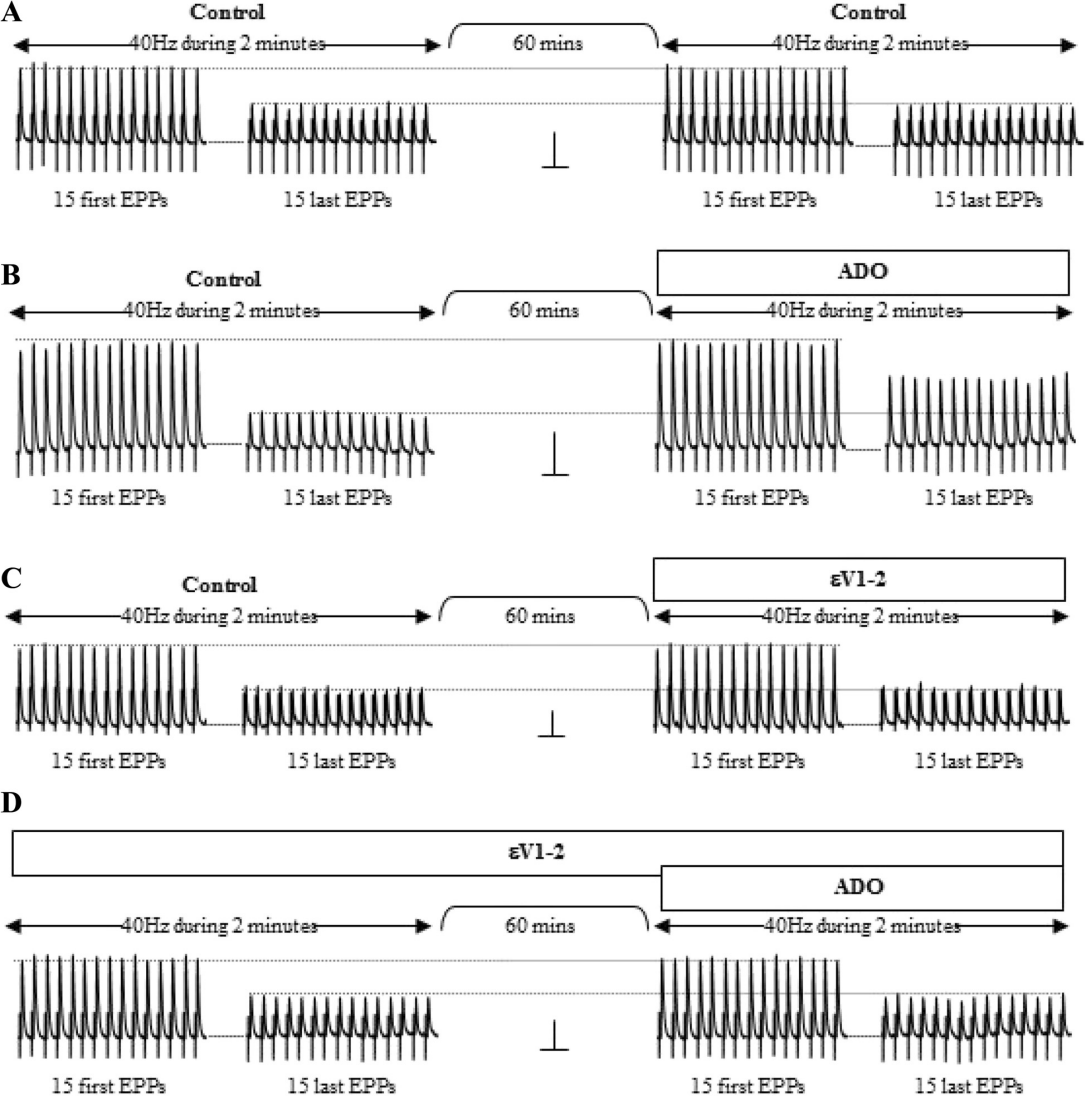
nPKCε and adenosine receptors in diaphragm muscles. We produced nerve-delivered stimulation (40 Hz, 2 min of supramaximal stimuli) and analyzed the effect of adenosine (ADO, 10 μM) and the peptide εV1-2 (10 μM) on modulating synaptic depression. We compared the mean size of the first 15 EPPs of each train and the mean size of the last 15 EPPs. The figure shows representative raw data. Horizontal bars: 50 ms. Vertical bars: 10 mV. **a** Effect of nerve-delivered stimulation (40 Hz, 2 min of supramaximal stimuli) and the normally occurring synaptic depression of the last EPPs in the train. **b** Effects of ADO in synaptic depression during imposed synaptic activity. **c** Effect of the peptide εV1-2. **d** Shows that, in the presence of εV1-2, added ADO cannot protect against depression

It seems that one of the major roles of adenosine receptors (AR) is to control synaptic depression. Synaptic depression during imposed synaptic activity (40Hz for 2 min of supramaximal stimuli reduce the EPP size by about 50 %, see Fig. 5a) is lessen by adding adenosine (Adenosine 5′-triphosphate disodium salt hydrate –ADO-, Fig. 5b) but increased by blocking AR with 8-(p-Sulfophenyl) theophylline hydrate (8SPT) (see [20, 21, 25]). We investigated the possible involvement of nPKCε in modulating synaptic depression during repetitive activity. We observed no difference in the size of the last EPPs in the presence and absence of the peptide εV1-2 (Fig. 5c). This suggests that the εV1-2 has no effect by itself on the normally produced activity-induced depression of the EPPs. Interestingly, however, in the presence of εV1-2, added ADO can not protect against depression (Fig. 5d). In the presence of εV1-2, AR are still tonically involved in the control of depression because the EPP size decreases as normal when receptors are blocked by 8SPT as occurs in the absence of the peptide (data not shown). These results suggest that nPKCε has a role in the purinergic mechanism of depression control. Whereas the tonic coupling of AR in the control of depression seems to be nPKCε independent, the kinase seems to be necessary if AR is to be additionally stimulated with ADO. The previous nPKCε translocation block using the peptide εV1-2 leads to the PKC uncoupling from ACh release in several conditions and also to the exogenous ADO being unable to protect against EPP depression.

## Discussion

Several PKC isoforms have been described in the presynaptic component of the NMJ [12, 33–39]: classical isoforms PKCα and PKCβI and the novel isoforms PKCθ and PKCε [12, 29]. Experiments on PKCθ knockout mice [39, 40] and PKCε block [29] suggest that these isoforms have a role in transmitter release. The main result of the present study is the observation that the nPKCε isoform helps to modulate transmitter release at the NMJ. Using the specific nPKCε translocation inhibitor peptide εV1-2 in electrophysiological experiments, we observed that the nPKCε was clearly involved in several conditions with the common denominator of PKC coupling to maintain or potentiate ACh release in the NMJ: i) low frequency electrical stimulation-induced activity, ii) high external Ca^2+^ and inflow, iii) PKA stimulation with Sp-8-BrcAMPs, iv) stimulation with phorbol ester and v) interference with mAChR-mediated presynaptic modulation of ACh release. In addition, we observed that nPKCε was involved in the AR control of synaptic depression.

The translocation inhibitor peptide εV1-2 has been used as a nonpharmacological tool in many studies with promising results. It interferes in the nPKCε interaction with the specific anchoring protein εRACK and, therefore, inhibits the anchoring of nPKCε near its substrates and prevents any subsequent substrate phosphorylation and activity [1]. εV1-2 does not interfere with classic calcium-dependent cPKCs not even with nPKCδ [1, 41–43]. Evidence also shows that the effects found using the nPKCε-specific translocation inhibitor peptide are confirmed when nPKCε knockout mice are used [44, 45]. In our experiments the effect of εV1-2 (100 μM) was studied in parallel to the effect of the scrambled version of this peptide (εV1-2-s, 100 μM). No effect of the inactive form was found. We also performed Western blot analysis to prove that the inhibitor peptide significantly decreases nPKCε levels in the synaptic membrane, which indicates that its translocation has been blocked. Furthermore, εV1-2 significantly also decreases pnPKCε levels in the synaptic membrane indicating a less amount of active nPKCε (pnPKCε) and, therefore, a decrease in the catalytical action of the nPKCε. We assayed εV1-2 concentrations of 1–100 μM. The range of values has been widely reported in the literature [45–50].

### PKC coupling to transmitter release

Now let us turn to the role of serine kinases in synaptic function. In resting neuromuscular preparations, PKA couples constitutively to ACh release [11]. However, although PKC can be stimulated pharmacologically with such phorbol esters as PMA to potentiate ACh release, PKC is uncoupled in basal conditions because quantal content does not change when all PKC isoforms are inhibited with the pan-inhibitor CaC [9–11]. However, taking CaC modification of the ACh release as a test, there are several experimental situations in which PKC isoforms play a regulated role in release modulation. In these situations, ACh release is increased and CaC reduces this release potentiation (which indicates that PKC is coupled to ACh release) [9–11, 51]. Quantal content is reduced by CaC incubation during continuous electrical stimulation at 1Hz [12], in the presence of high external Ca^2+^ [9], after PKA stimulation with Sp-8-BrcAMP (as example of intracellular pathways modification [11]) and after mAChR block [10, 51]. When nPKCε translocation is blocked with the peptide εV1-2, the PKC coupling to ACh release (evidenced by CaC incubation) cannot be demonstrated in electrical stimulation and high Ca^2+^ conditions and ACh release potentiation does not occur in PKA stimulation with Sp-8-BrcAMPs or in mAChR block. Likewise, after nPKCε translocation block, PKCs cannot be stimulated with PMA and exogenous adenosine cannot work against repetitive activity-induced depression. This isoform, then, may be involved in the presynaptic function of maintaining and potentiating transmitter release, probably by controlling the coupling of other PKC isoforms to the ACh release.

Interestingly, in all cases, the nPKCε translocation to the membrane needs to be blocked some time before (typically the preincubation with εV1-2 takes 1 h) the onset of the changes in the conditions that lead to PKC coupling. It seems that once the nPKCε translocation to the membrane has been triggered by, for instance, phorbol ester stimulation, 1 Hz trains or muscarinic signaling collapse with AT, the PKC isoform activation makes the synapse potentiation-competent for some time, such that subsequent incubation with εV1-2 produces no effect. A long-lasting permanence of phosphorylated nPKCε in the membrane, the permanence of the phosphorylated PKC substrates or a cascade of events initiated during the initial kinase activation may produce this potentiation competent state. Recent studies have demonstrated that PKC phosphorylates several molecules of synaptic vesicle exocytic apparatus and there is evidence that these PKC-mediated phosphorylations contribute directly to the regulation of the neurotransmitter release [52–54]. These proteins may be the link between the ACh release machinery and nPKCε.

When nPKCε translocation is blocked after PKC has coupled to ACh release (in all the experimental conditions studied here), there is no change in ACh release, which indicates that nPKCε is not able to modulate neurotransmission once the release mechanism has been activated. However, quantal content is reduced by CaC in the same conditions, which indicates that other PKC isoforms may continue the initial permissive effect of nPKCε on release potentiation.

**Fig. 6:**
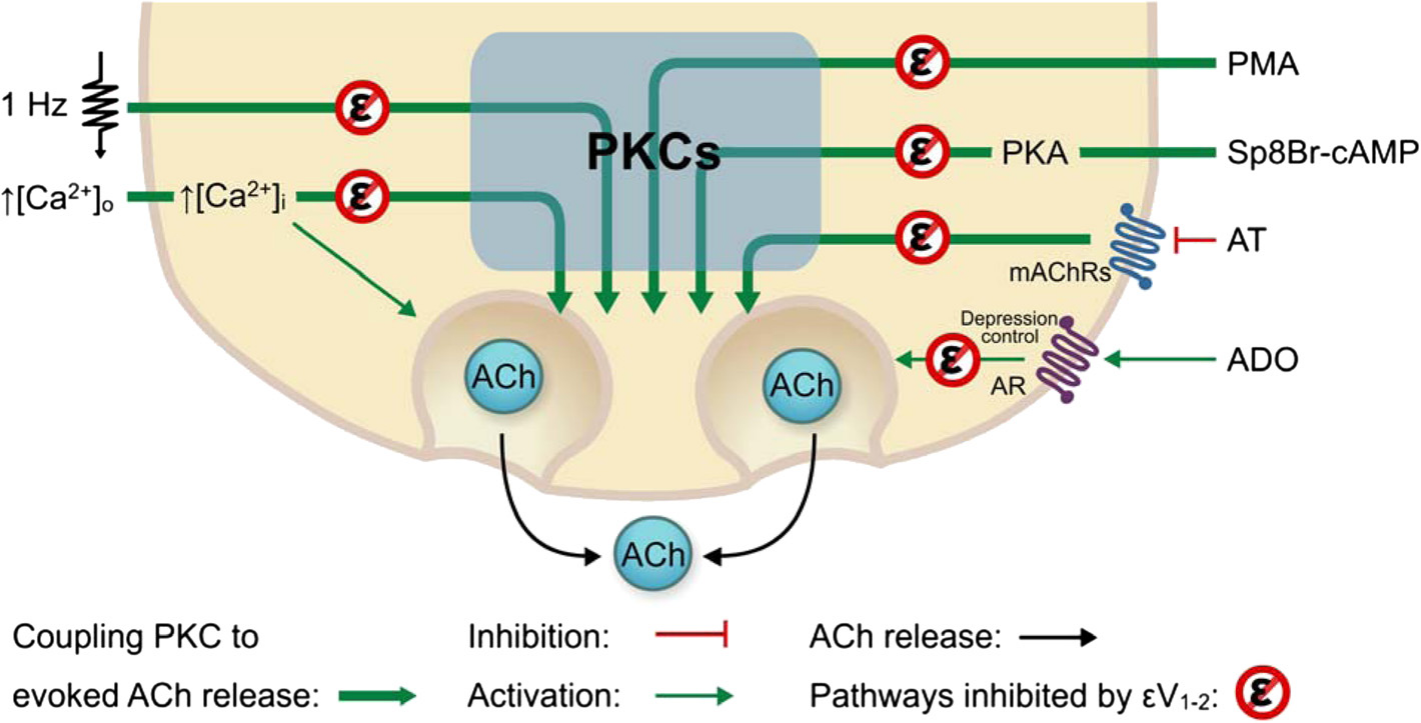
Graphic representation of the central role of nPKCε activity in the regulated coupling of PKC to ACh release potentiation. The diagram illustrates several conditions that are known to couple PKC to enhance ACh release in the NMJ: electrical stimulation (1 Hz), high Ca^2+^ inflow, stimulation with phorbol ester (PMA), PKA stimulation with Sp-8-BrcAMP, and the interference with presynaptic mAChR (with atropine –AT–) and AR (with adenosine –ADO–). In all these conditions, preincubation with the specific nPKCε translocation inhibitor peptide εV1-2 impairs the PKC coupling and highlights the nPKCε isoform role in the modulation of ACh release in this synapse

Therefore, although the specific role of nPKCε in ACh release is not known, here we show that it is involved in a crucial step in the release process. The individual analysis of each condition may provide additional insight into particular aspects of nPKCε involvement. Figure 6 is a graphic representation of the main observations of this study, showing that the blockade of nPKCε translocation, and therefore its activity, impedes the regulated coupling of PKC to ACh release potentiation.

### nPKCε and electrical stimulation

Blocking nPKCε translocation prevents electrical stimulation from coupling PKCs to ACh release. However, electrical stimulation by itself (at 1 Hz) does not change quantal content [12] and the present results show that neither does blocking the nPKCε translocation by itself. Thus, the CaC-inhibitable and nPKCε translocation-dependent PKC coupling to ACh release during electrical stimulation and continuous activity may be involved in some maintenance (or sustainability) function but not in direct ACh release potentiation. However, the tonic maintenance effect of the PKC coupling and the PMA-induced coupling, which result in release potentiation, share a common nPKCε link because the PMA effect is fully prevented with a lower concentration of εV1-2 if it coincides with electrical stimulation.

### nPKCε and Ca^2+^ions

Blocking the epsilon isoform prevents PKC coupling in high Ca^2+^ concentration, which helps to potentiate release. Thus, also in this case, nPKCε translocation and PKC coupling to ACh release seems to be closely related. Because nPKCε is a Ca^2+^-independent isoform, nPKCε would be activated and coupled to ACh release by diacylglycerol in the context of the exocytotic process started by Ca^2+^ entry.

### nPKCε and PKA stimulation

PKA stimulation with Sp-8-BrcAMP results in Ca^2+^-dependent increased ACh release and a parallel CaC-inhibitable PKC coupling [10, 11]. Preincubation with εV1-2 prevents Sp-8-BrcAMP release potentiation. This indicates that at least some of the PKA-mediated potentiation of release may be produced by the involvement of nPKCε. As shown here, blocking PKCε with the specific blocker εV1-2 does not prevent the inhibitory effect of H-89, which indicates that PKA is tonically active and, therefore, that PKA can modulate transmitter release to a certain level independently of nPKCε status. Interestingly, however, after εV1-2 incubation, PKA cannot be stimulated with Sp-8-BrcAMPs. Thus, nPKCε translocation inhibition may be enough to prevent PKA from functioning above its basal tonic activity.

### nPKCε and the PMA stimulation of PKC

All PKC isoforms can be stimulated pharmacologically by phorbol esters such as PMA, which also increase ACh release and need Ca^2+^ ions [9]. However, CaC cannot revert the PMA effect to the initial point, although it can be suppressed by preincubation with CaC. This shows that PMA and CaC are powerful positive and negative irreversible pharmacological regulators of PKC activity.

We investigate how blocking nPKCε translocation affects the PMA effect. PMA cannot enhance ACh release when εV1-2 is present at high concentration (100 μM) or when εV1-2 is present only at 10 μM but coincides with electrical stimulation at 1Hz. Thus, the effect of PMA on neurotransmission is largely dependent on nPKCε, and the PMA and electrical activity mechanisms partially share a common pathway.

In previous studies we identified several conditions that hamper or prevent the PMA-induced stimulation of PKC coupling to potentiate ACh release just as happens with εV1-2 preincubation. What do these conditions have in common that can shed some light on the role of nPKCε? PMA cannot stimulate release after blocking (H-89) or stimulating (Sp-8-BrcAMP) PKA and after reducing (5 mM Mg^2+^, μ-Agatoxin) or increasing (5 mM Ca^2+^) Ca^2+^ inflow [11]. After PKA is stimulated or Ca^2+^ inflow increased, PKCs are active and coupled to release potentiation. We believe that the PKC pathway may be saturated almost to full capacity (in relation to ACh release) so it would not be additionally activated by PMA. On the contrary, after PKA is blocked or Ca^2+^ inflow reduced, PKCs are inactive and uncoupled to release so the pathway may be inactive or blocked. Thus, with respect to the well known phorbol ester effect, it seems that blocking nPKCε translocation mimics blocking PKA activity or reducing Ca^2+^ inflow. These data reinforce the notion that there is a close relation between nPKCε, PKA and Ca^2+^ ions in the promotion of ACh release and strongly suggest that nPKCε plays a central role in transmitter release.

Interestingly, some experimental conditions prevent the PMA effect while others allow it. PMA is able to increase ACh release during or after electrical stimulation or when the mAChRs are blocked [10]. This suggests that in these two conditions other PKC isoforms may also be involved in ACh release (see later).

Altogether the results suggest that the translocation of PKCε to the membrane is necessary if PKC family is to be involved in ACh release in the NMJ. In turn, this suggests that this isoform plays a key permissive role that may allow other PKCs to have a positive effect on ACh release. It seems that nPKCε is key to (and may be causally involved in) the high ACh release situations described here: namely, high Ca^2+^ entry, continuous synaptic activity, PKA stimulation and phorbol ester stimulation of PKCs.

### nPKCε and the mAChR pathway

In the presynaptic membrane, mAChRs are an important self-control mechanism of ACh release [10, 15–18, 55–57]. In the NMJ, the intracellular coupling of the PKC pathway can mediate the mAChR modulation of ACh release at least in part [10]. Specifically, blocking the muscarinic mechanism results in a CaC-inhibitable PKC coupling and ACh release potentiation. Thus, PKC become coupled on mAChR signaling inhibition. We found here that after an initial incubation with εV1-2, a second incubation with AT, PIR or MET produces no change in EPP size or quantal content. These data suggest that, in basal conditions, mAChRs reduce PKC activity and ACh release. Impairing the muscarinic function results in PKC coupling and increased release, in which nPKCε seems to play an important role. Thus, the muscarinic modulation of release can be prevented by PKCε translocation block.

Interestingly, as stated in the section above, PMA can increase ACh release after the action of the blockers (AT, PIR, MET). Thus, the effects of AT and PMA seems to be additive. The PKC isoforms activated by the use of AT are probably not all isoforms and this allows PMA to complete the activation of the remaining isoforms and the same may occur with the electrical stimulation and PMA effects that are also additive as previously stated. Thus, nPKCε could be involved in activity- and muscarinic-dependent mechanisms of release modulation together with other PKC isoforms.

### nPKCε and adenosine receptors signaling

Exogenous added ADO reduces synaptic depression at a moderate level of imposed activity (40Hz) on the NMJ. At high levels of activity (100Hz), endogenous ADO production in the synaptic cleft can be sufficient to interact with A_1_R receptors to protect against depression [20, 21, 25]. Here we found that in the presence of εV1-2, added ADO is not able to protect against depression. If nPKCε is an important element in the adenosine-mediated mechanism of depression control because it increases the quantal content of the last EPPs in a train (the coupling of PKCs in the adult NMJ potentiates ACh release), the nPKCε translocation inhibitor peptide εV1-2 must prevent the protective effect on depression of ADO, as we found here.

## Conclusion

In summary, by blocking nPKCε translocation to the membrane in electrophysiological experiments, we show a clear involvement of this PKC isoform in several conditions with the common denominator of PKC coupling to maintain or potentiate ACh release in the NMJ. These conditions are: electrical stimulation, high Ca^2+^ inflow, stimulation with phorbol ester, PKA stimulation, and interference with presynaptic mAChR and AR. In all these conditions, preincubation with the specific nPKCε translocation inhibitor peptide εV1-2 impairs PKC isoforms coupling to ACh release and points to the nPKCε isoform as a key element in the modulation of ACh release in this synapse. These results are represented in Fig. 6.

## Methods

### Animals

Diaphragm muscles of young adult male Sprague–Dawley rats (30–40 days; Criffa, Barcelona, Spain) were used to perform stimulation experiments, Western blotting and electrophysiological experiments. Diaphragm and LAL muscles were used to perform immunohistochemistry. The animals were cared for in accordance with the guidelines of the European Community Council Directive for the humane treatment of laboratory animals. This study was approved by the Ethics Committee of the Rovira i Virgili University (Ref. number 233).

### Antibodies

The primary antibodies used for Western blot and immunohistochemistry analysis were obtained from the following sources: rabbit anti-PKCε and goat anti-phospho-PKCε (Ser729) polyclonal antibodies from Santa Cruz Biotechnology (Santa Cruz, CA); rabbit anti-PKCε and rabbit anti-phospho-PKCε (Ser729) polyclonal antibodies from Upstate Biotechnology (Millipore, Lake Place NY); goat anti-GAPDH (glyceraldehyde 3-phosphate dehydrogenase) from Imgenex (San Diego, CA) and rabbit anti-pan-actin polyclonal antibody from Cell Signaling Technology, Inc (Beverly, MA). The secondary antibodies used in the Western blot were donkey anti-rabbit conjugated to HRP (Horseradish Peroxidase) from Jackson Immunoresearch and rabbit anti-goat HRP from Molecular Probes (Eugene, OR). For the immunohistochemistry we also used antibodies that are commonly used as markers to differentially detect the parts of the NMJ (syntaxin and S100): mouse monoclonal and rabbit polyclonal anti-syntaxin antibodies (Sigma, St Louis, MO); rabbit anti-S100 antibody (Dako, Carpinteria, CA) and mouse anti-S100 antibody (Acris, Germany). The secondary antibodies used were donkey anti-rabbit or donkey anti-mouse conjugated to Alexa Fluor 488 and Alexa Fluor 647 from Molecular Probes (Eugene, OR). Postsynaptic acetylcholine receptors (AChRs) were detected with α-bungarotoxin (α-BTX) conjugated to tetramethyl rhodamine iso-thiocyanate (TRITC) from Molecular Probes (Eugene, OR).

As a control, the primary antibodies were omitted from some muscles during the immunohistochemical and Western blot procedures. None of these control muscles exhibited positive staining or revealed bands of the appropriate molecular weight with the respective procedures. In double-staining protocols, omitting either one of the two primary antibodies completely abolished the corresponding staining and there was no cross-reaction with the other primary antibody. Pretreatment of a primary antibody with an excess of the appropriate blocking peptide (between three- and eightfold by weight) in skeletal muscle tissue prevented immunolabeling. All the primary antibodies detected a single band with the referenced molecular weight on Western blot (manufacturer’s data sheets; [29]).

### Reagents

For the different treatments we used substances that modulate ACh release involving PKC activity.

#### nPKCε-specific translocation inhibitor peptide

εV1-2, nPKCε-specific translocation inhibitor peptide (myristoylated PKC-ε V1-2 peptide, EAVSLKPT) from Calbiochem (Merk, Germany) was made up as 1 mM in distilled water or normal Ringer solution. Working solutions were 1, 10 and 100 μM. As a negative control of the nPKCε-specific translocation inhibitor peptide we used scrambled εV1-2 peptide (εV1-2-s, LSETKPAV), containing the same aminoacids as the inhibitor peptide but in a different sequence, from Calbiochem (Merk, Germany). In no case does εV1-2-s have an effect.

#### Drugs that modulate PKC activity

Phorbol 12-myristate 13-acetate (PMA, Sigma) was made up as a 10 mM stock solution in dimethylsulfoxide (DMSO, Tocris, Ellisville, MO, USA). Calphostin C (CaC, Sigma) was made up as a 2.5 mM stock solution in DMSO. Working solutions were PMA, 10nM and CaC, 10 μM.

#### Calcium, magnesium and P/Q-type calcium channel blocker

In some experiments, we increased the content of calcium or magnesium in the bath to 5 mM. The *P/Q-type calcium channel blocker*, the toxin ω-Agatoxin IVA (ω-Aga-IVA), was purchased from Research Biochemicals Inc. Controls and toxin-treated muscles were assayed in the presence of 0.01 % bovine serum albumin (BSA) (Sigma, St. Louis, MO, USA). Working solutions of ω-Aga-IVA are 100 nM.

#### Muscarinic agents

Stock solutions: Pirenzepine dihydrochloride 10 mM (PIR, Tocris), Methoctramine tetrahydrochloride 1 mM (MET, Sigma) and Atropine 200 μM (AT, Sigma). Working solutions: PIR 10 μM, MET 1 μM, and AT 2 μM.

#### Drugs that modulate PKA activity

N-[2-((p-Bromocinnamyl)amino)ethyl]-5-isoquinolinesulfonamide, 2HCl (H-89, Calbiochem) was made up as a 5 mM stock solution in DMSO. Adenosine 3′,5′-cyclic Monophosphorothioate, 8-Bromo-, Rp-Isomer, Sodium Salt (Sp-8-BrcAMPs, Calbiochem) was made up as a 5 mM stock solution in deionized water. Working solutions were Sp-8-BrcAMPs 10 μM and H-89 5 μM.

#### Nonselective AR agonist

The stock solution of Adenosine 5′-triphosphate disodium salt hydrate (ADO, Sigma-Aldrich) was made up as a 100 mM solution in deionized water. The working solution was 10 μM.

All stock solutions were stored at −20 °C for less than four weeks. We chose drug concentrations that did not change the size of the MEPPs in the concentration-response curves performed in previous experiments. The final DMSO concentration in control and drug-treated preparations was 0.1 % (v/v). In control experiments, this concentration of DMSO did not affect any of the parameters studied (data not shown).

### Stimulation of the muscle and incubations with reagents

In all the experimental protocols, the diaphragm muscle from young adult rats were excised together with the phrenic nerve and placed in oxygenated Ringer solution (see below) continuously bubbled with 95 % O2 / 5 % CO_2_ at room temperature. To stimulate the muscle, the phrenic nerve was stimulated at 1 Hz by an A-M Systems 2100 isolated pulse generator (A-M System, Carlsborg, WA). Muscle contraction was abolished by using μ-conotoxin GIIIB (μ-CgTx-GIIIB, 3 μM −1.5 μM, from ICS, International Clinical Service GmbH, München).

Consecutive incubations with two substances are used as a pharmacological tool to investigate the possible occlusive or additive crosstalk effects between them. We recorded and measured control EPPs, and then incubated the muscle for one hour in the first compound. After recording the EPPs again, we incubated it for one hour in the second compound (in the presence of the first drug) and then recorded the EPPs.

### Western blot analysis

Diaphragm muscles from adult rat were dissected, frozen in liquid nitrogen, and stored at −80 °C before use. The muscles were homogenized using a high-speed homogenizer (overhead stirrer, VWR International, Clarksburg, MD) in lysis buffer containing 150 mM NaCl, 20 mM Tris–HCl, pH 7.5, 2 mM EGTA (Ethylene Glycol Tetraacetic Acid), and 5 mM EDTA (Ethylenedinitrilo-tetraacetic acid) supplemented with 1 % Triton X-100, 1 mM PMSF (phenylmethanesulfonyl fluoride), 50 mM NaF, and 1 mM sodium orthovanadate from Sigma, (St. Louis, MO) and protease inhibitor cocktail (Sigma-Aldrich Corp., Saint Louis, MO, USA). Insoluble material was removed by centrifugation at 1000 g for 10 min. The supernatants were collected and centrifuged at 15000 g for 20 min. Finally, the resulting supernatants (total protein lysates) were collected. Protein concentrations were determined by using the Bio-Rad DC protein assay (Bio-Rad, Hercules, CA). Experimental procedures were performed to determine the linear and quantitative dynamic range for each target protein and the appropriate dilutions of samples were used for accurate and normalized quantitation by means of densitometric analysis. Protein samples of 15 or 30 μg were separated by 8 % SDS-polyacrylamide electrophoresis and electrotransferred to polyvinylidene difluoride (PVDF) membranes (Hybond^TM^-P; Amersham, GE Healthcare). The membranes were blocked in Tris-buffered saline-0.1 % Tween-20 (TBS-T) containing 5 % (W/V) nonfat dry milk or in a blocking reagent to preserve phosphoprotein antigens (PhosphoBLOCKER™; Cell Biolabs, Inc.) and probed with the primary antibody overnight at 4 °C. The membranes were then incubated with the secondary antibody and visualized the enhanced chemiluminescence with the ECL kit (Amersham Life Science, Arlington Heights, IL).

In diaphragm muscles, the synaptic membranes were obtained. Synaptic and extrasynaptic parts of the diaphragm muscle were separated as previously described [12]. We performed control experiments to check that our protocol for dividing the diaphragm muscle into synaptic and extrasynaptic region was accurate. In some muscles, we repeated the process of separation and detected NMJs with TRITC-conjugated α-BTX. We also stained the nerves with an antibody against anti-neurofilament-200 and did not detect any nerves in extrasynaptic regions. The muscles were homogenized using a high-speed homogenizer (overhead stirrer, VWR International, Clarksburg, MD) in lysis buffer (see above) and the insoluble material was removed in the same way (by centrifugation at 1000 g for 10 min) but this time the resulting supernatant was collected and centrifuged at 130000 g for 1 h. The supernatant was the cytosolic fraction, and the pellet was the membrane fraction. To assess the separation of the membrane fraction from the cytosol, we used a goat antibody directed against GAPDH, a protein specific to the cytosolic fraction. GADPH immunoreactivity was not observed in any case in the membrane fraction. The samples were processed the same way as another sample of total protein (see below).

The blots were visualized with a VersaDoc 3000 (Bio-Rad, Hercules, CA). The densitometry of the bands was analyzed with the MetaMorph software. The integrated optical density of the bands was normalized by actin protein and to the background values. Also, as another loading control, we used a total protein analysis (Sypro Ruby protein blot Stain, Bio Rad) to measure the total protein transferred on PVDF membranes. In all cases, the quantitative results obtained by using actin or total protein analysis were no different. The relative variations between the bands in the experimental samples and the control samples were calculated from the same image. The data were taken from densitometry measurements made in at least five separate experiments, plotted against controls. Data are mean values ± SD. Differences between groups were tested using the t Student test or U test (Mann–Whitney), and the normality of the distributions was tested with the Kolmogorov-Smirnov test. The criterion for statistical significance was *p* < 0.05 versus the control.

### Immunohistochemistry and confocal microscopy

Whole muscle mounts were processed by immunohistochemistry to detect the localization of the nPKCε isoform at the NMJ. LAL and diaphragm muscles were used to perform the immunohistochemistry technique. Muscles from young adult rats were fixed with 4 % paraformaldehyde for 30 min. After fixation, the muscles were rinsed with PBS and incubated in 0.1 M glycine in PBS. The muscles were permeabilized with 0.5 % Triton X-100 in PBS, and nonspecific binding was blocked with 4 % bovine serum albumin (BSA). Then, muscles were incubated overnight at 4 °C in mixtures of three primary antibodies raised in different species (anti-nPKCε isoform antibody and anti-syntaxin or anti-S100) and rinsed. The muscles were then incubated for four hours at room temperature in a mixture of secondary antibodies. The AChRs were detected with α-BTX conjugated with TRITC. At least three muscles were used as negative controls as described above. For a better analysis of the localization of the nPKCε isoform at the NMJ, muscles were processed to obtain semithin cross-sections from whole-mount multiple-immunofluorescent stained muscles. This method provided a simple and sensitive procedure for analyzing the cellular distribution of molecules at the NMJ [32].

Labeled NMJs from the whole-mount muscles and the semithin cross-sections were viewed with a laser-scanning confocal microscope (Nikon TE2000-E). Special consideration was given to the possible contamination of one channel by another. In experiments involving negative controls, the photomultiplier tube gains and black levels were identical to those used for a labeled preparation made in parallel with the control preparations. There were no differences in nPKCε immunolocalization between diaphragm and LAL muscles. Since better NMJ images can be obtained from LAL, we decided to perform a wider study on this muscle. At least 25 endplates per LAL muscle were observed, and at least six muscles were studied. Images were assembled using Adobe PhotoShop software (Adobe Systems, San Jose, CA) and neither the contrast nor the brightness was modified.

### Electrophysiology

Diaphragm muscles from adult rats and their nerve supply were surgically removed and pinned in a Sylgard-lined 35-mm Petri dish containing normal Ringer solution (in mM) – NaCl 135, KCl 5, CaCl_2_ 2.5, MgSO_4_ 1, NaH_2_PO_4_ 1, NaHCO_3_ 15, glucose 11 – and bubbled continuously with 95 % O_2_, 5 % CO_2_, which flowed into the Petri dish to superfuse the muscle preparation. The overflow was evacuated by suction. The solution was not bubbled directly in the Petri dish to minimize vibration during electrophysiological recording. Temperature and humidity were set to 26 °C and 50 %, respectively. The bath temperature was monitored during the experiments (23.4 °C ± 1.7, Digital Thermometer TMP 812, Letica, Barcelona, Spain). Intracellular recordings (EPPs and MEPPs) were performed with conventional glass microelectrodes filled with 3 M KCl (resistance: 20–40 MΩ). Recording electrodes were connected to an amplifier (Tecktronics, AMS02, Oregon, USA), and a distant Ag-AgCl electrode connected to the bath solution via an agar bridge (agar 3.5 % in 137 mM NaCl) was used as reference. The signals were digitized (DIGIDATA 1322A Interface, Axon Instruments Inc., Weatherford, TX, USA), stored and computer-analyzed. The software Axoscope 9.0 (Axon Instruments Inc.) was used for data acquisition.

To prevent muscle contraction during EPP recordings, we used μ-CgTx-GIIIB(1.5 μM) with a recirculation system. After a muscle fiber had been impaled, the nerve was continuously stimulated (70 stimuli at 0.5Hz) using two platinum electrodes coupled to a pulse generator (CIBERTEC CS-20) and linked to a stimulus isolation unit. We recorded the last 50 EPPs. We selected fibers with membrane potentials of no less than -70 mV and used only those results from preparations which did not deviate by more than 5 mV during the recording. The mean amplitude (mV) per fiber was calculated and corrected for non-linear summation (EPPs were usually more than 4 mV) [58] assuming a membrane potential of – 80 mV. Quantal content (M) was estimated by the direct method, which consists of recording MEPPs and EPPs simultaneously and then calculating the ratio: M = Average Peak EPP/Average Peak MEPP. Incubation with the drugs took place for one hour. We studied a minimum of 15 fibers per muscle and usually a minimum of 5 muscles in each type of experiment.

We also applied repetitive stimulation (trains at 40 Hz for 2 min) to evaluate the effects of the peptide εV1-2 and reagents affecting adenosine receptors on synaptic depression. There were 10-min intervals between trains to allow for muscle recovery. We recorded 2 min of EPPS and used the first 15 and the last 15 EPPS to evaluate changes in depression. We evaluated the ratio between the mean size of the last 15 EPPs of each train and the mean size of the first 15 EPPs. We also analyzed possible facilitation as the ratio between the sizes of the second EPP and the first EPP in each train of 40Hz. We studied between 8 and 10 fibers per muscle and usually between 5 and 8 muscles in each type of experiment. In the single-fiber experiments, the drugs were added to the bathing solution and the EPPs were recorded every 15 min for 60 min.

Standard sharp-electrode intracellular recording techniques were used to show that MEPP amplitudes and postsynaptic resting membrane potentials were unaffected and, therefore, that all the compounds used act presynaptically in the present conditions. The MEPP frequency in each solution was recorded for 100 s from at least 15 different neuromuscular junctions and the values were averaged. ACh in the synaptic cleft can increase during trains of 40 Hz. This may modify the sensitivity of the AChRs. Therefore, we evaluated the size of the MEPPs during the trains and did not notice any change with the drugs used. For example, the change in the amplitude of MEPPs during trains of 40Hz in the presence of 25 μM adenosine was 14.90 % ± 2.07 and for 8-SPT it was 2.41 % ± 4.65 (P < 0.05).

The statistical software SPSS© v17.0 was used to analyze the results. Values are expressed as means ± SEM. The values are expressed as “percentage of change”. This is defined as: [final value / initial value] X 100. We used Welch’s two-tailed t-test for unpaired values because our variances were not equal. We prefer this test because it is more conservative than the ordinary t-test. Differences were considered significant at P < 0.05.

## Abbreviations

PKC: Protein kinase C
RACKs: Receptors for activated C-kinase
NMJ: Neuromuscular junction
PKA: Protein kinase A
mAChR: Presynaptic muscarinic acetylcholine autoreceptors
NR: Neurotrophin receptors
ACh: Acetylcholine
nPKCε: Protein kinase C epsilon
εV1-2: nPKCε-specific translocation inhibitor peptid
PMA: Phorbol 12-myristate 13-acetate
Sp-8-BrcAMP: Adenosine 3′,5′-cyclic Monophosphorothioate, 8-Bromo-, Rp-Isomer, Sodium Salt
pnPKCε: Phosphoprotein kinase C epsilon
LAL: Levator auris longus muscle
nAChR: Nicotinic acetylcholine receptor
EPP: Endplate potentials
MEPPs: Miniature endplate potentials
CaC: Calphostin C
AT: panmuscarinic blocker atropine
VDCC: voltage-dependent calcium channels
ω-Aga-IVA: toxin ω-Agatoxin IVA
−89: N-[2-((p-Bromocinnamyl)amino)ethyl]-5-isoquinolinesulfonamide, 2HCl
PIR: M1 mAChR-subtype blocker pirenzepine
MET: M2 blocker methoctramine
AR: Adenosine autoreceptors
ADO: Adenosine 5′-triphosphate disodium salt hydrate
8-SPT: 8-(p-Sulfophenyl) theophylline hydrate
AChR: Acetylcholine receptor
α-BTX: α-bungarotoxin
TRICT: Tetramethyl rhodamine iso-thiocyanate
DMSO: Dimethylsulfoxide
BSA: Bovine serum albumin
μ-CgTx-GIIIB: μ-conotoxin GIIIB
PVDF: Polyvinylidene difluoride
TBST: Tris-buffered saline Tween 20
GAPDH: Glyceraldehyde 3-phosphate dehydrogenase
